# An Evaluation of the Left-Brain vs. Right-Brain Hypothesis with Resting State Functional Connectivity Magnetic Resonance Imaging

**DOI:** 10.1371/journal.pone.0071275

**Published:** 2013-08-14

**Authors:** Jared A. Nielsen, Brandon A. Zielinski, Michael A. Ferguson, Janet E. Lainhart, Jeffrey S. Anderson

**Affiliations:** 1 Interdepartmental Program in Neuroscience, University of Utah, Salt Lake City, Utah, United States of America; 2 Departments of Pediatrics and Neurology, University of Utah, Salt Lake City, Utah, United States of America; 3 Department of Bioengineering, University of Utah, Salt Lake City, Utah, United States of America; 4 Waisman Laboratory for Brain Imaging and Behavior, Department of Psychiatry, Division of Child & Adolescent Psychiatry, University of Wisconsin, Madison, Wisconsin, United States of America; 5 Department of Radiology, University of Utah, Salt Lake City, Utah, United States of America; 6 The Brain Institute at the University of Utah, Salt Lake City, Utah, United States of America; Beijing Normal University, China

## Abstract

Lateralized brain regions subserve functions such as language and visuospatial processing. It has been conjectured that individuals may be left-brain dominant or right-brain dominant based on personality and cognitive style, but neuroimaging data has not provided clear evidence whether such phenotypic differences in the strength of left-dominant or right-dominant networks exist. We evaluated whether strongly lateralized connections covaried within the same individuals. Data were analyzed from publicly available resting state scans for 1011 individuals between the ages of 7 and 29. For each subject, functional lateralization was measured for each pair of 7266 regions covering the gray matter at 5-mm resolution as a difference in correlation before and after inverting images across the midsagittal plane. The difference in gray matter density between homotopic coordinates was used as a regressor to reduce the effect of structural asymmetries on functional lateralization. Nine left- and 11 right-lateralized hubs were identified as peaks in the degree map from the graph of significantly lateralized connections. The left-lateralized hubs included regions from the default mode network (medial prefrontal cortex, posterior cingulate cortex, and temporoparietal junction) and language regions (e.g., Broca Area and Wernicke Area), whereas the right-lateralized hubs included regions from the attention control network (e.g., lateral intraparietal sulcus, anterior insula, area MT, and frontal eye fields). Left- and right-lateralized hubs formed two separable networks of mutually lateralized regions. Connections involving only left- or only right-lateralized hubs showed positive correlation across subjects, but only for connections sharing a node. Lateralization of brain connections appears to be a local rather than global property of brain networks, and our data are not consistent with a whole-brain phenotype of greater “left-brained” or greater “right-brained” network strength across individuals. Small increases in lateralization with age were seen, but no differences in gender were observed.

## Introduction

Lateralized brain regions direct functions such as language and visuospatial processing. In most right-handed individuals, paying attention to stimuli involving language elicits brain activity lateralized to the left hemisphere, whereas paying attention to stimuli involving visuospatial processing elicits brain activity lateralized to the right hemisphere [1]–[4]. Atypical lateralization in brain structure and function is associated with neuropsychiatric disorders such as autism spectrum disorders and schizophrenia [5]–[10], although there is considerable variation within typically developing individuals in the strength to which specific functions such as language are lateralized to the canonical side, particularly for left-handed and ambidextrous individuals [11].

Previous studies of brain laterality are largely limited to regional assessment of specialized functions and differences in structural lateralization. It has been well documented that small structural asymmetries consisting of a frontal (right>left) and occipital (left>right) shear effect are present in most individuals [12], in addition to asymmetries of the planum temporale, angular gyrus, caudate, and insula [13]. A diffusion tensor study of a predefined brain parcellation using graph-theoretical methods showed increased efficiency and connectedness within the right hemisphere, but with regions of greatest network centrality in the left hemisphere [14]. Additional asymmetries in gray matter volume have been observed within nodes of the default mode network [15].

With the recent development of resting state functional connectivity magnetic resonance imaging (rs-fcMRI) techniques, it has become possible to characterize whole-brain lateralization using a data-driven approach. Two recent studies have investigated whole-brain lateralization using rs-fcMRI [16], [17]. Liu et al. (2009) found that connectivity of classical language regions, medial prefrontal cortex, and posterior cingulate cortex was most strongly left-lateralized, whereas that of insula, angular gyrus, anterior cingulate cortex, and visual cortex was most strongly right-lateralized. Males had more strongly lateralized connections than females. In a factor analysis, the four factors that accounted for the most variance involved regions from the following cortical networks: visual, default, salience, and language. Handedness influenced the laterality of the four factors; however, it affected laterality differently across the factors.

Tomasi and Volkow (2012) demonstrated that short- and long-range connections were predominantly right-lateralized in brain regions surrounding the lateral sulcus, whereas left-lateralized connections were limited to medial areas of the occipital cortex and superior rim of the parietal and posterior frontal lobes [17]. Additionally, much of the medial aspect of the frontal and parietal lobes had right-lateralized long-range connections, whereas Broca Area and angular gyrus had left-lateralized long-range connections. As in Liu et al. (2009), males had more lateralized connections than females, although the effect was small.

These studies raise important questions. Does functional connectivity lateralization reflect structural asymmetry or does it represent a lateralized difference in the strength of synaptic connections? Does a whole-brain phenotype of relatively greater “left-brain” or “right-brain” functional specialization across individuals exist, or are lateralized connections in different brain networks independent of each other within an individual? Are these connectivity patterns modified with age, as the brain matures into an adult phenotype? In this manuscript, we address these questions and find that lateralized regions create left- and right-lateralized networks, lateralized connections are independent from one another across individuals, and that the majority of functional lateralization occurs before age seven.

## Materials and Methods

### Publicly Released Datasets –1011 Subjects

1011 subjects were analyzed from publicly available datasets released with the open-access 1000 Functional Connectomes Project (http://fcon_1000.projects.nitrc.org/) in which resting-state functional magnetic resonance imaging (fMRI) scans have been aggregated from 28 sites [18] as well as typically developing subjects from the ADHD 200 project from the International Neuroimaging Data-sharing Initiative (fcon_1000.projects.nitrc.org/indi/adhd200/index.html) including 8 sites [19]. For inclusion we required that subjects’ ages were between 7 and 29, with BOLD whole-brain coverage from Montreal Neurologic Institute (MNI) coordinates z = −35 to z = 70. Any subject for whom preprocessed data did not cover all 7266 regions of interest (ROIs) used for this analysis was discarded prior to analysis (see Anderson et al. [20] for a list of the MNI coordinates for the 7266 ROIs). Also for inclusion, all subjects included a magnetization-prepared rapid acquisition with gradient echo (MPRAGE) anatomic sequence that was successfully segmented and normalized to MNI space. Although preprocessing steps were performed using an automated batch script, the results of normalization, segmentation, and realignment steps were manually inspected for all subjects, and any subject for whom the normalized and segmented images were not in close alignment with the MNI template on visual inspection were discarded. The datasets from which subjects met all criteria are listed in Table 1. The mean age of all subjects was 18.3+/−5.6 s.d. years (range 7–29). 587 subjects were male; 424 were female. All subjects were processed in the same manner regardless of the site from which they were obtained.

**Table 1:** Sources of open access datasets used for analysis of 1011 scans.

Site (FCON 1000)	n (Imaging Volumes)	Site (FCON 1000)	n (Imaging Volumes)	Site (ADD 200)	n (Imaging Volumes)
Ann Arbor	16 (295)	Leipzig	29 (195)	Kennedy Krieger	49 (124*)
Baltimore	11 (123)	New York	30 (192*)	NeuroImage	18 (261)
Bangor	1 (265)	Newark	15 (135)	NYU	87 (352*)
Beijing	187 (225)	Orangeburg	3 (165)	OHSU	22 (234)
Berlin	16 (195)	Oulu	33 (245)	Peking	109 (236)
Cambridge	171 (119)	Oxford	8 (175)	Pittsburgh	72 (196*)
Cleveland	5 (127)	Palo Alto	6 (175)	Washington U	35 (396*)
ICBM	13 (128)	Queensland	14 (190)		
Leiden	30 (215)	Saint Louis	31 (127)		

* Sites with multiple runs or sequences with differing numbers of imaging volumes. The reported number of imaging volumes is the most frequently used number per subject for the site.

### Gray Matter Density Measurements and Structural Lateralization Metric

Gray matter density images were created by normalizing and segmenting MPRAGE images using SPM8 (Wellcome Trust, London) into three tissue classes representing gray matter, white matter, and cerebrospinal fluid (CSF). Smoothly varying intensity changes as well as artifactual intensity alterations as a result of the normalization step were corrected for using a standard modulation algorithm within SPM. We then derived mean gray matter intensities within 7266 spherical (5 mm radius) seed ROI [20]–[22] that formed a lattice covering the gray matter.

Segmented gray matter images from the normalized MPRAGE images were also flipped across the midsagittal plane, and the difference in mean gray matter density was recorded for each ROI as the structural lateralization index ([unflipped density - flipped density]/[unflipped density+flipped density]).

### fMRI Preprocessing

The following sequence was used for image preprocessing of all blood oxygen level-dependent (BOLD) image datasets. Using SPM8 toolbox (Wellcome Trust, London), BOLD images were realigned (realign, estimate and write), coregistered to MPRAGE image (coregister, estimate and write), and the MPRAGE image (with coregistered BOLD images) was normalized to an MNI template with spatial resolution of 3 mm^3^ voxels (normalize, estimate and write, T1.nii template). Gray matter, white matter and CSF were segmented from MPRAGE image using SPM8 segment function (modulated, normalized, thorough clean). Images were bandpass filtered between 0.001 and 0.1 Hz and a linear detrend was performed at each voxel in the brain. The lower limit of 0.001 Hz was chosen in order to be certain as much neural information was included as possible [23]. The linear detrend removed much of the contribution of low frequencies given the relatively short time series available in the dataset. Time series were averaged from two ROIs in the white matter (bilateral centrum semiovale), CSF (lateral ventricles), soft tissues of the head and face, and six rigid motion correction parameters from realignment step as previously described and for each voxel [24], a general linear model was used to find a best fit for white matter, CSF, soft tissues, and motion parameter time series, which were subtracted from the voxel’s time series. No regression of the global signal was included. No smoothing was performed to avoid contaminating the signal near the midsagittal plane. Recent reports have highlighted the necessity to take extra precaution when dealing with motion artifact [25]–[27]. Therefore, a motion scrubbing procedure was implemented that involved removing frames with DVARS or root-mean-square motion parameters >0.2 mm prior to analysis of connectivity results [27].

### Functional Lateralization Metric

Functional correlation was obtained as the Fisher-transformed Pearson correlation coefficient between each pair of the 7266 ROIs within the same hemisphere. We only analyzed connections within a single hemisphere and the opposite hemisphere homologues because of ambiguity of “lateralization” of a cross-hemisphere connection. Preprocessed images were inverted across the midsagittal plane, and analogous Fisher-transformed correlation coefficients were obtained between each pair of the same ROIs on the flipped images. Functional lateralization index was defined as the difference (unflipped - flipped) between Fisher-transformed correlation coefficients. The functional lateralization index did not include the normalization term in the denominator like the structural lateralization index or that is commonly used in functional lateralization studies [28] because the functional connectivity correlations include positive and negative values rather than strictly positive values. The use of a denominator when calculating a functional lateralization index may result in index values with a discontinuity in the denominator, binary index values (e.g., if flipped = −0.01 and unflipped = +0.01, then [unflipped – flipped]/[|unflipped|+|flipped|] = 1), or index values that accentuate small differences in laterality (e.g., if flipped = 0.01 and unflipped = 0.03, then [unflipped – flipped]/[|unflipped|+|flipped|] = 0.5). Moreover, the functional correlation measurements already occupy the interval between −1 and 1.

**Figure 1:**
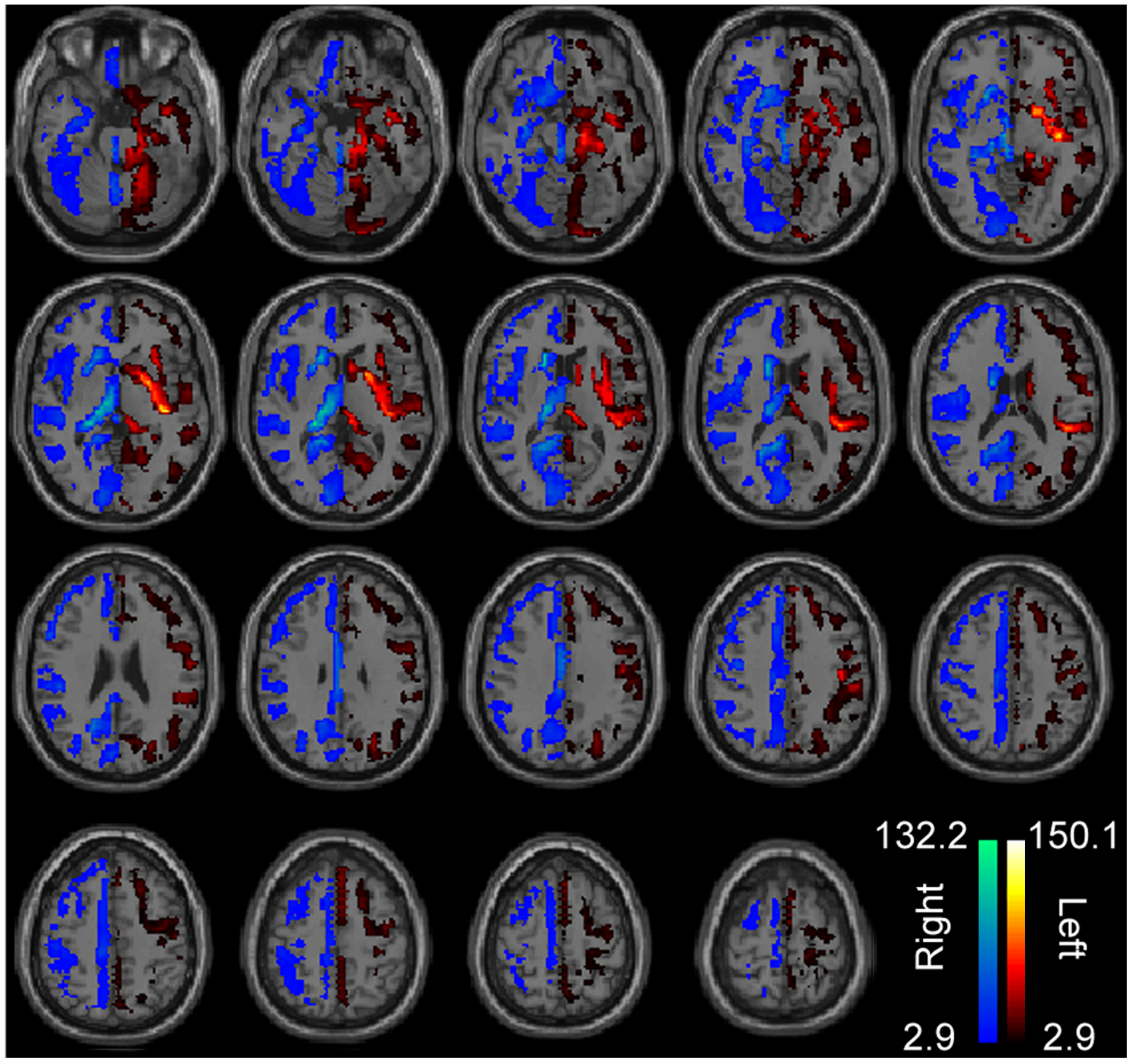
Significant lateralization of gray matter density. Colored regions included ROIs that showed significantly greater left- or right-lateralization of gray matter density across 1011 subjects, correcting for multiple comparisons using a false discovery rate correction of q<0.05 across 7266 ROIs. Color bars show t-statistics for the left and right hemispheres, respectively. Images are in radiologic format with subject left on image right.

The structural effects were regressed out of the functional lateralization metrics. For each of the 7266 ROIs, the structural lateralization indices (Figure 1) calculated for the given ROI and the other 7265 ROIs were regressed from the corresponding functional lateralization indices on a subject-by-subject basis using a general linear model (glmfit.m in MATLAB). More specifically, for a connection involving two ROIs, the mean structural lateralization index for the two ROI endpoints was used as a regressor, with regression performed across the set of all connections for an individual subject. Most of the structural/functional correlation was removed after regression, although a residual relationship remains. These data indicate that even after accounting for subject-to-subject variation in structural asymmetries, nodes that show more gray matter in one hemisphere tend to have stronger functional connections involving that node in the same hemisphere.

**Figure 2:**
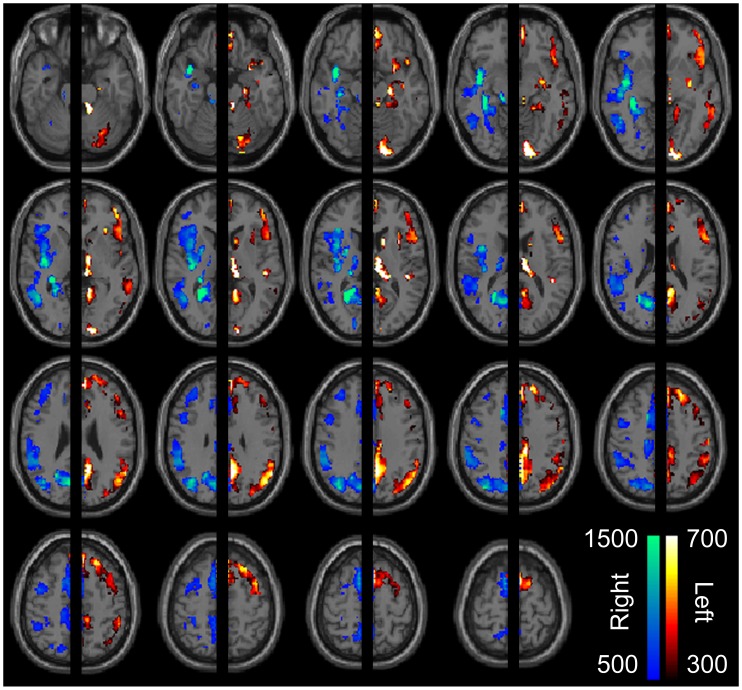
Degree maps for significantly left- and right-lateralized connections after regression of structural laterality index from all connections. Significantly lateralized connections (after correcting for multiple comparisons using a false discovery rate of q <0.05, across all 14.1 million intrahemispheric connections) were used to construct a graph of significantly left-lateralized connections among left hemisphere ROIs and a separate graph of significantly right-lateralized connections among right hemisphere ROIs. Color scale shows graph-theoretical degree (i.e., sum of all significantly lateralized connections in which a given node is represented) for each ROI. Images are in radiologic format with subject left on image right.

After regression, significantly lateralized connections were those for which a two-tailed t-test showed values that were different from 0 after correction for multiple comparisons using acceptable false discovery rate of q<0.05. Sparse binarized graphs of significantly left- and right-lateralized connections were obtained and degree was calculated as the sum of all significantly left- or right-lateralized connections in which a given node is represented. Hubs were defined as local maxima in the images of degree of the left- and right-lateralized graphs (Table 2 and Figure 2). In neuroimaging literature, it is common to refer to hubs as brain regions that are highly connected, either structurally or functionally, to other brain regions and play a central role in brain network dynamics [29]–[31]. In this manuscript, we take that definition one step further by referring to hubs as brain regions that are involved in many lateralized functional connections. Thus, “hubs” need not represent nodes of intrinsic connectivity networks. Large changes in degree were seen with structural regression compared to without structural regression in the occipital pole, medial posterior insula, caudate, putamen, thalamus, and lingual gyrus adjacent to the occipital horn of the lateral ventricle. These regions were not considered hubs in subsequent analyses since there was likely a large effect of structural asymmetry on lateralization. We identified 9 remaining hubs in the left-lateralized graph and 11 hubs in the right-lateralized graph. We ensured that all 9 left-lateralized hubs and 11 right-lateralized hubs, respectively, were at least 10 mm apart from one another. Two of the left hubs were within 10 mm of the interhemispheric homologues of two of the right hubs (Broca Area and Broca Homologue and left and right supplementary motor area), meaning the areas participate in strongly lateralized connections in both hemispheres.

**Table 2:** MNI coordinates of 20 lateralization hubs.

Left Hemisphere Hubs	X	Y	Z	Right Hemisphere Hubs	X	Y	Z
Broca Area (Br)	−45	25	0	Right Supplementary Motor Area (r-S)	5	8	61
Wernicke Area (We)	−58	−44	−2	Mid Insula (MI)	38	4	12
Inferior Dorsolateral Prefrontal Cortex (DP)	−43	43	1	Parietooccipital (PO)	36	−74	35
Left Supplementary Motor Area (l-S)	−6	10	62	Lateral Intraparietal Sulcus (LI)	55	−44	32
Lateral Premotor Cortex (LP)	−35	8	53	Frontal Eye Fields (FE)	43	0	51
Medial Prefrontal Cortex (MP)	−4	51	19	Dorsolateral Prefrontal Cortex (DL)	34	40	32
Medial Superior Frontal (SF)	−16	34	46	Middle Temporal Area (MT)	49	−60	0
Posterior Cingulate Cortex (PC)	−4	−56	31	Broca Homologue (Bh)	43	26	−3
Lateral Temporoparietal Junction (TP)	−45	−67	30	Mid Cingulate Cortex (MC)	13	−32	47
				Superior Medial Intraparietal Sulcus (IP)	12	−73	44
				Anterior Insula (AI)	36	26	8

### Statistical Analyses

All statistical analyses were performed in MATLAB using MATLAB’s statistical toolbox. Each cortical hub’s lateralization pattern with other hubs in the ipsilateral hemisphere of the cerebral cortex was determined by performing one-sample t-tests on the functional connections involving the cortical hub as the seed and the other ipsilateral hubs. Global versus local lateralization was tested by calculating a functional lateralization index for connections involving right-hemispheric hubs (i.e., 11 right-hemispheric hubs resulting in 55 pairwise connections) and connections involving left-hemispheric hubs (i.e., 9 left-hemispheric hubs resulting in 36 pairwise connections) for each subject and then covarying each connection with all other connections across subjects for a total of 4095 pairs of 91 connections. This effectively asks whether two connections, each between hubs in one hemisphere, tend to be relatively stronger in the same subjects. To test for gender effects, two-sample t-tests were applied to 1) the average left and right functional laterality index values for each subject and 2) on the set of connections involving the 20 lateralization hubs (total of 195 comparisons). To test the effects of age, correlations were measured for 1) the average left and right functional laterality index values for each subject and 2) the set of connections involving the 20 lateralization hubs (total of 195 correlations).

To test whether the results from a single site corresponded with overall results, the mean functional laterality indices for the Beijing (site with largest sample size) subjects were correlated with the mean functional laterality indices for all other subjects. To test if excessive noise was introduced by including sites with small samples (i.e., <10 subjects), the mean functional laterality indices excluding the 23 subjects from sites with small samples were correlated with the mean functional laterality indices for all subjects. Spearman correlations (because the non-Gaussian nature of the data) were used to test whether there was any relationship between functional lateralization index of the 91 connections involving intrahemispheric hubs and the following movement measurements: mean movement during scan, maximum movement from one frame to the next, the number of frames discarded during the scrubbing procedure described above, and the percent of frames discarded during the scrubbing procedure. All analyses in this manuscript that involved more than a single test included a correction for multiple comparisons using a false discovery rate of q <0.05.

## Results

**Figure 3:**
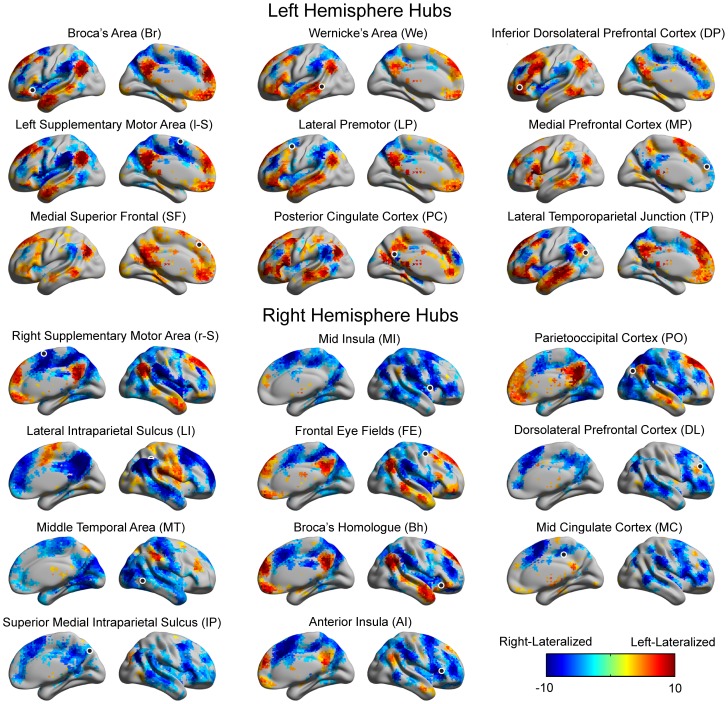
Significantly lateralized connections to each hub. The hemispheric lateralization maps for the nine hubs of the left-lateralized network and 11 hubs of the right-lateralized network are shown in lateral and medial projections. Color scale (t-statistic) shows significantly left-lateralized (warm colors) or right-lateralized (cool colors) to the seed (i.e., hub). A black circle marks the position for each seed.

We first investigated each cortical hub’s lateralization pattern across the ipsilateral hemisphere of the cerebral cortex. The lateralization pattern consisted of two parts (Figure 3). First, the left-lateralized connections included regions from the default mode network (medial prefrontal cortex, posterior cingulate cortex, temporoparietal junction, and inferior temporal cortex) and classical language regions (Broca Area and Wernicke Area). Second, the right-lateralized connections included regions that can be broadly categorized as attentional areas (frontal eye fields, area MT, anterior cingulate cortex, insular cortex, supplementary motor area, intraparietal sulcus, superior parietal lobules, and dorsolateral prefrontal cortex). The lone exception among the left-hemispheric hubs, the medial prefrontal cortex, shared right-lateralized connections with much of the typically left-lateralized surrounding cortex and the posterior cingulate cortex. Among the right-hemispheric hubs there were two patterns: hubs that were right-lateralized to DMN (and all other right-hemispheric hubs), and hubs that were left-lateralized to DMN (but right-lateralized to the right-hemispheric hubs). Nevertheless, some of the hubs that were right-lateralized (such as lateral IPS) to all 20 hubs show extensive left-lateralized connections to non-hub regions, indicating that lateralization networks have hub-specific features.

**Figure 4:**
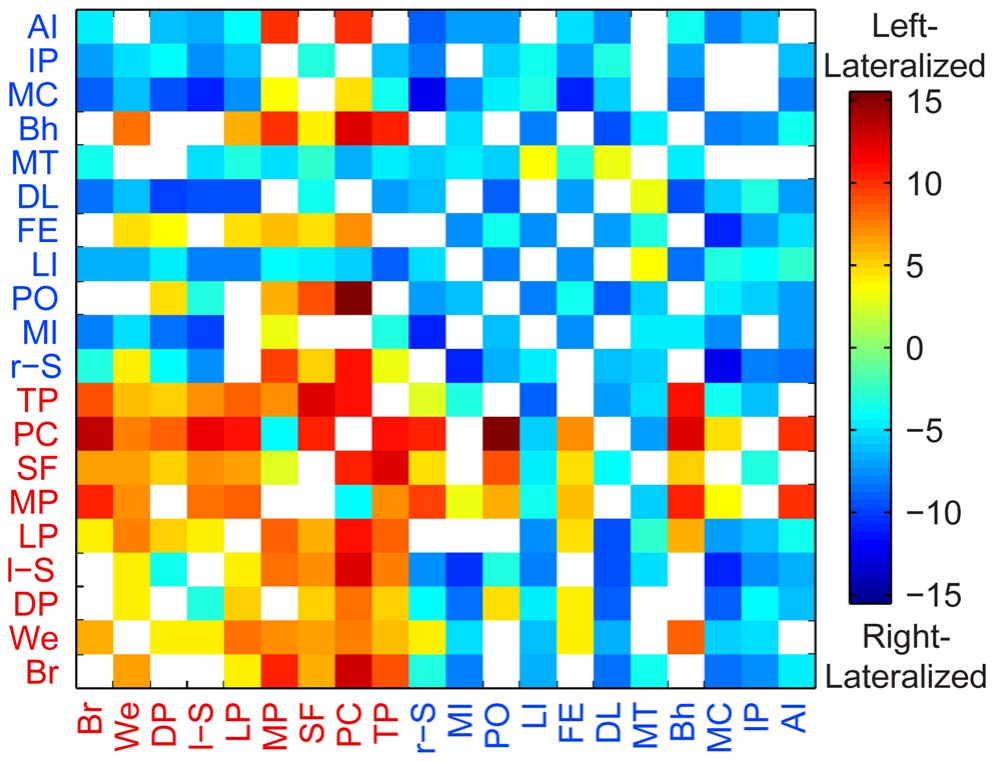
Significantly lateralized connections between each of the 20 hubs. Warm colors show significant left lateralization and cool colors show significant right lateralization. Color bar shows t-statistic for each connection. All colored squares were significant after correcting for multiple comparisons using a false discovery rate of q<0.05 among all possible connections between the hubs. See Table 2 or Figure 3 for the hubs’ two-letter abbreviations.

The laterality of connections between the 20 hubs is summarized in Figure 4. Colored squares indicate connections where the functional lateralization index, after regression of the structural lateralization index across subjects, was significantly left or right lateralized after FDR correction for multiple comparisons across all possible connections among the 20 hubs. When comparing the laterality between interhemispheric connections (i.e., connection involving a left-lateralized hub and a right-lateralized hub), the functional lateralization index was calculated by flipping the right-lateralized hub across the midsagittal plane into the left hemisphere in order to maintain intrahemispheric comparisons. Connections between left-hemispheric hubs were almost entirely left-lateralized, and connections between right-lateralized hubs were almost entirely right-lateralized. Although the hubs were selected for having a high degree in the graph of significantly lateralized connections, this did not require the hubs to all show consistent lateralization with each other and suggests that the left-hemispheric hubs and right-hemispheric hubs form a backbone of two broader lateralized networks in the brain, one in the left hemisphere and one in the right hemisphere.

**Figure 5:**
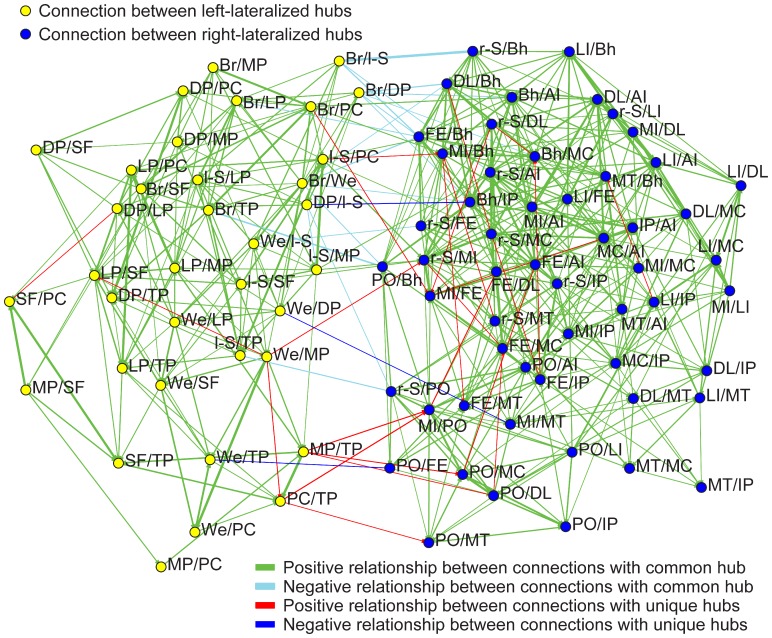
Significant correlation of lateralized connections across subjects. Yellow nodes represent connections between left hubs and green nodes represent connections between right hubs. An edge is present if lateralization was found to significantly correlate across subjects between the two connections, with red edges showing positive correlation and blue edges negative correlation, after correcting for multiple comparisons using a false discovery rate of q<0.05 across all possible connection-to-connection pairs. Virtually all edges are between nodes with a hub in common. A Kamada-Kawai algorithm was implemented in Social Network Image Animator software (http://www.stanford.edu/group/sonia/). The software was also used to visualize the relationship between connections. See Table 2 or Figure 3 for the hubs’ two-letter abbreviations.

Next, we determined whether lateralization was a whole brain or a local property. In other words, if connections between left-hemispheric hubs were strongly left-lateralized in a subject, did this correspond to connections among right-hemispheric hubs showing stronger right lateralization? Figure 5 summarizes the results. Of the 630 comparisons involving left-hemispheric hubs, only one (0.2%) showed significant negative correlation (i.e., as one connection between left-hemispheric hubs became more left-lateralized the other connection between left-hemispheric hubs became less left-lateralized), whereas 144 significant comparisons (22.9%) involved positively correlated connections. Of the 990 comparisons involving right-hemispheric hubs, none negatively correlated and 329 comparisons (33.2%) involved positively correlated connections. Almost all of the significant positively correlated connections (left: 141/144; right: 314/329) included connections with a common hub. Of the 1,620 comparisons involving right-hemispheric hub connections versus left-hemispheric hub connections, 20 were significantly negatively correlated (1.2%) and 16 are significantly positively correlated (1.0%). The majority of the significant negatively correlated connections (16/20) and significant positively correlated connections (8/16) included connections with a right-hemispheric hub that when flipped across the midline is <10 mm from a left-hemispheric hub.

Together, these results imply lateralization is a local property rather than a whole-brain property. If a hub formed a strongly lateralized connection with another ipsilateral hub in a subset of subjects, it was more likely that the same hub would form strongly lateralized connections with other ipsilateral hubs in those subjects. But with rare exceptions, no effect was seen between other distinct ipsilateral hubs in the same subjects.

**Figure 6:**
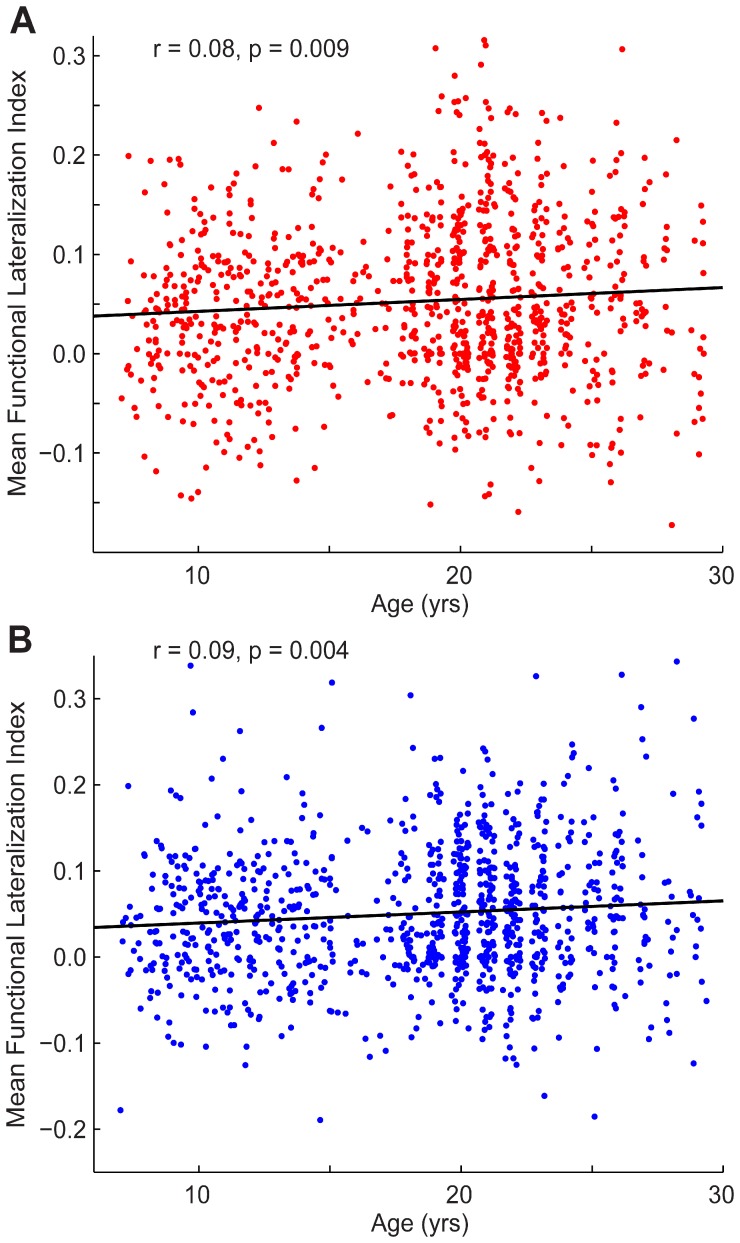
Change in mean functional lateralization with age. Mean functional lateralization index for all connections between left **(A)** and right **(B)** hubs, respectively, is shown for each subject, plotted against subject age. Pearson correlation coefficients and p-values are shown above both plots.

We investigated the effects of gender on lateralization and how lateralization changes over development between the ages of 7 and 29. No significant gender effects were found when testing the mean lateralization for the connections involving left- and right-hemispheric hubs, respectively, or a subset of connections between the 20 hubs. We found small, significant relationships between age and mean lateralization for the connections involving left- and right-hemispheric hubs, respectively (Figure 6; left: *r* = 0.08 *p* = 0.009; right: *r* = 0.09 *p* = 0.004). Because there was a significant effect, albeit small, when averaging across all connections between left-hemispheric or right-hemispheric hubs, we extended our analysis to the individual left hub-left hub and right hub-right hub connections. Table 3 lists the ten right-lateralized connections that become significantly more right-lateralized across development and survive correction for multiple comparisons using a false discovery rate of q <0.05.

**Table 3:** Connections between right-lateralized hubs that change in lateralization across development between the ages of 7 and 29.

Hub 1	Hub 2	r	p
Right Supplementary Motor Area	Mid Insula	0.129	5.7e-5
Right Supplementary Motor Area	Middle Temporal Area	0.089	0.0052
Right Supplementary Motor Area	Mid Cingulate Cortex	0.084	0.0092
Mid Insula	Broca Homologue	0.116	0.0003
Parietooccipital	Frontal Eye Fields	0.099	0.0021
Parietooccipital	Mid Cingulate Cortex	0.110	0.0006
Lateral Intraparietal Sulcus	Broca Homologue	0.102	0.0013
Frontal Eye Fields	Middle Temporal Area	0.083	0.0087
Frontal Eye Fields	Mid Cingulate Cortex	0.088	0.0063
Frontal Eye Fields	Superior Medial Intraparietal Sulcus	0.128	6.9e-5

**Figure 7:**
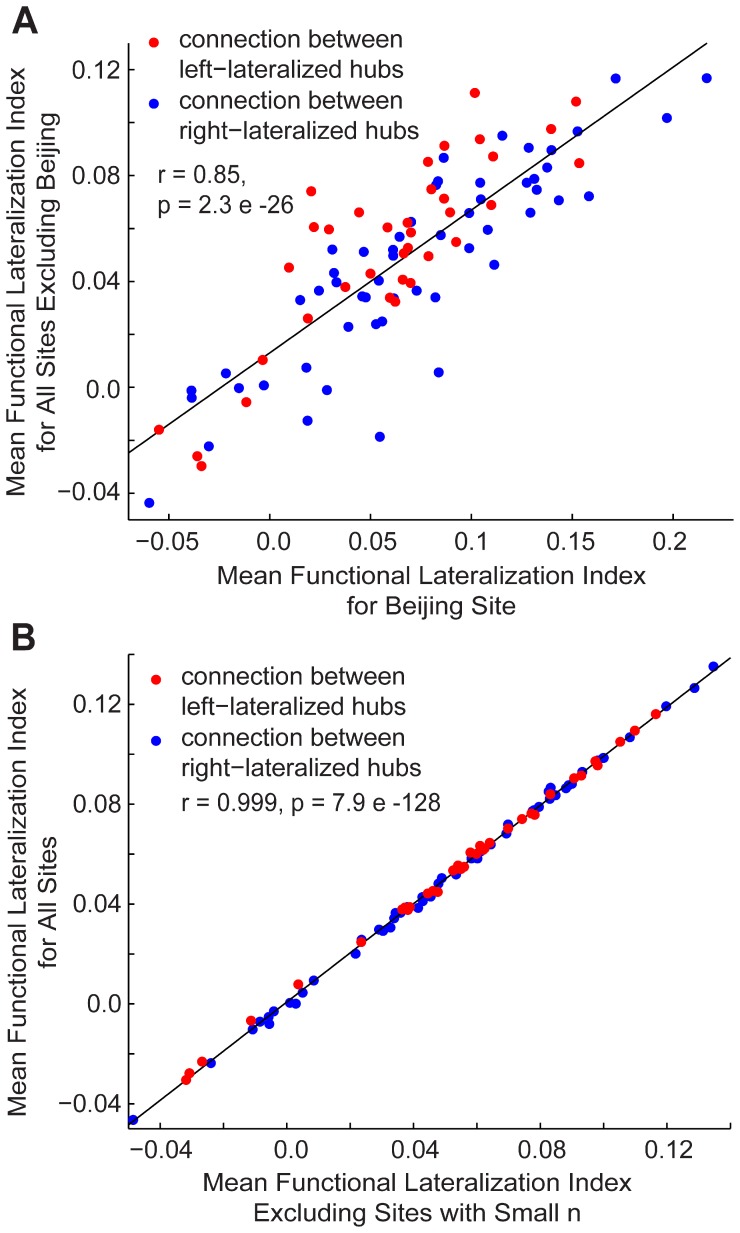
Reproducibility of lateralization. **A**, Mean functional lateralization index for the 91 intrahemispheric connections (blue, connections involving right-lateralized hubs; red, connections involving left-lateralized hubs) is compared when averaging across all subjects except those from the Beijing site and when averaging across only subjects from the Beijing site. Pearson correlation coefficients and p-values are shown in both plots. **B**, Mean functional lateralization index for the 91 intrahemispheric connections (blue, connections involving right-lateralized hubs; red, connections involving left-lateralized hubs) is compared when averaging across all subjects and when averaging across all subjects except those that come from a site with less than 10 subjects.

Finally, we tested whether the results described were reproducible in a smaller sample and whether they were due to potential confounds. We compared the relationship between mean functional lateralization of the 91 connections involving intrahemispheric hubs from the Beijing site, the site with the largest sample size, and the mean from all other sites. The measurements between the two subsamples corresponded highly (*r = *0.85, *p* = 2.3 e-26). We also determined that including sites with small samples (five sites with less than 10 subjects for a total of 23 subjects) did not introduce excessive amounts of variability (Figure 7B) and that the lateralization results were not due to head motion artifact. The mean functional lateralization of the 91 connections involving intrahemispheric hubs was virtually identical when including subjects from sites with small samples (Figure 7B; *r = *0.999, *p* = 7.9 e-128). No relationship between the functional lateralization index of the 91 connections involving intrahemispheric hubs and the single-subject motion measurements (e.g., mean movement, the number of frames discarded during the scrubbing procedure described above, etc.) survived multiple comparison correction (false discovery rate of q <0.05).

## Discussion

By comparing the magnitude of functional connectivity in a large multi-site cohort (n = 1011) of subjects, we demonstrate that a left-dominant network and a right-dominant network can be defined in which discrete hubs show consistent lateralization among connections between the respective left- and right-hemispheric hubs. The identified left-dominant and right-dominant hubs correspond well to known architecture of intrinsic connectivity networks, and show persistent lateralization of connectivity even after removal of the variance attributed to structural asymmetry of gray matter. We also demonstrate that lateralization is a local rather than a whole-brain property. In other words, when a connection of interest is strongly lateralized, the degree of lateralization for the other connections throughout the brain relates only in the connections that have a hub in common with the connection of interest.

Our data is broadly consistent with previous studies regarding the spatial distribution of lateralization of functional connectivity [16], [17]. We find that brain regions showing consistently strong left-lateralization include classical language regions (Broca Area, Wernicke Area, lateral premotor, and anterior supplementary motor areas). MNI coordinates associated with greatest left-lateralization match closely those reported in task-based fMRI studies of language [32]. Broca and Wernicke Areas have been shown to comprise a distributed language network, predominantly left-lateralized, in their functional connections and include both adjacent cortical as well as subcortical regions [33].

Other left-lateralized hubs include core regions of the default mode network (posterior cingulate, medial prefrontal, temporoparietal junction) [34]. In a diverse assortment of cognitive tasks [35], this network shows greater activity during the resting state than during the task [36], and it has been proposed that this network may be involved in attending to internal stimuli, internal narrative, or self-reflection [37]–[40]. Recent evidence suggests this network may be comprised of a midline core active during self-referential thought, and a medial temporal core active during memory of past events [41], with the precuneus showing three anterior/posterior subdivisions with differing connectivity patterns [42].

In contrast, hubs of right-lateralized functional connectivity correspond well to canonical regions of the dorsal and ventral attention networks and the cingulo-insular or salience network [43]–[47]. This network is more active during tasks requiring attention to external stimuli or assessment of stimulus salience or novelty [46], [48]. Virtually all of the described hubs of this network show right lateralization to each other in our analysis, including intraparietal sulcus, frontal eye fields, area MT, anterior insula, and dorsolateral prefrontal cortex. Right lateralization of external stimulus attention is consistent with lesion studies reporting much greater incidence of hemispatial neglect following right-hemispheric injury [49], particularly associated with lesions to regions of the ventral attention network [49].

In popular reports, “left-brained” and “right-brained” have become terms associated with both personality traits and cognitive strategies, with a “left-brained” individual or cognitive style typically associated with a logical, methodical approach and “right-brained” with a more creative, fluid, and intuitive approach. Based on the brain regions we identified as hubs in the broader left-dominant and right-dominant connectivity networks, a more consistent schema might include left-dominant connections associated with language and perception of internal stimuli, and right-dominant connections associated with attention to external stimuli.

Yet our analyses suggest that an individual brain is not “left-brained” or “right-brained” as a global property, but that asymmetric lateralization is a property of individual nodes or local subnetworks, and that different aspects of the left-dominant network and right-dominant network may show relatively greater or lesser lateralization within an individual. If a connection involving one of the left hubs is strongly left-lateralized in an individual, then other connections in the left-dominant network also involving this hub may also be more strongly left lateralized, but this did not translate to a significantly generalized lateralization of the left-dominant network or right-dominant network. Similarly, if a left-dominant network connection was strongly left lateralized, this had no significant effect on the degree of lateralization within connections in the right-dominant network, except for those connections where a left-lateralized connection included a hub that was overlapping or close to a homotopic right-lateralized hub.

We observe that lateralization of uncorrected functional correlation measurements includes a significant effect from structural asymmetries such as gyral position. We attempted to correct for this effect by regressing out gray matter density across subjects for each of the endpoints of every connection in our dataset to obtain a less biased measurement of functional lateralization. Although this effect is difficult to completely remove, it is unlikely that the relationships we describe are wholly attributable to structural asymmetries. The map of gray matter density lateralization shows a different spatial distribution from the map of functional connectivity lateralization, with structural lateralization varying abruptly between left and right with each gyrus, and functional lateralization following well-known functional architecture of intrinsic connectivity networks. Two of the nodes are within 10 mm of their homotopic equivalents in the left- and right-dominant networks. Thus, the same hub is lateralized to one set of connections in the left hemisphere and to a different set of connections in the right hemisphere. This is consistent with prior diffusion tensor and functional connectivity MRI analyses showing that connections between the temporoparietal junction and insula are asymmetrically lateralized to the right, while connections between the temporoparietal junction and the inferior frontal gyrus are asymmetrically lateralized to the left [50], [51].

It is also possible that the relationship between structural lateralization and functional lateralization is more than an artifact. Brain regions with more gray matter in one hemisphere may develop lateralization of brain functions ascribed to those regions. Alternately, if a functional asymmetry develops in a brain region, it is possible that there may be hypertrophy of gray matter in that region. The extent to which structural and functional asymmetries co-evolve in development will require further study, including imaging at earlier points in development and with longitudinal imaging metrics, and whether asymmetric white matter projections [52], [53] contribute to lateralization of functional connectivity.

It is important to note that our data measure only asymmetries in the magnitude of functional connectivity between homotopic connections, but do not measure differences in the content of cognitive information between analogous connections in opposite hemispheres. Thus, a connection in the left hemisphere could be associated with a completely novel neural computation from a homotopic connection in the right hemisphere yet show no difference in functional connectivity lateralization. Nevertheless, lateralized functional correlation suggests a network architecture that differs between the two hemispheres and may be an indicator of the content of the two networks given known differences in function of the respective left- and right-lateralized hubs.

We observed a weak generalized trend toward greater lateralization of connectivity with age between the 20 hubs included in the analysis, but most individual connections did not show significant age-related changes in lateralization. The weak changes in lateralization with age should be interpreted with caution because the correlations included >1000 data points, so very subtle differences may be observed that are not associated with behavioral or cognitive differences. Prior reports with smaller sample sizes have reported differences in lateralization during adolescence in prefrontal cortex [54] as well as decreased structural asymmetry with age over a similar age range [55].

Similarly, we saw no differences in functional lateralization with gender. These results differ from prior studies in which significant gender differences in functional connectivity lateralization were reported [16], [17]. This may be due to differing methods between the two studies, including the use of short-range connectivity in one of the former reports and correction for structural asymmetries in this report. A prior study performing graph-theoretical analysis of resting state functional connectivity data using a predefined parcellation of the brain also found no significant effects of hemispheric asymmetry with gender, but reported that males tended to be more locally efficient in their right hemispheres and females tended to be more locally efficient in their left hemispheres [56].

It is intriguing that two hubs of both the left-lateralized and right-lateralized network are nearly homotopic. Maximal left-lateralization in Broca Area corresponds to a similar right-lateralized homotopic cluster extending to include the anterior insula in the salience network. Although both networks have bilateral homologues in the inferior frontal gyrus/anterior insular region, it is possible that the relative boundaries of Broca Homologue on the right and the frontoinsular salience region may “compete” for adjacent brain cortical function. Future studies in populations characterized for personality traits [57] or language function may be informative as to whether local connectivity differences in these regions are reflected in behavioral traits or abilities. The study is limited by the lack of behavioral data and subject ascertainment available in the subject sample. In particular, source data regarding handedness is lacking. However, none of the hubs in our left- and right- lateralized networks involve primary motor or sensory cortices and none of the lateralized connections showed significant correlation with metrics of handedness in subjects for whom data was available.

Despite the need for further study of the relationship between behavior and lateralized connectivity, we demonstrate that left- and right-lateralized networks are homogeneously stronger among a constellation of hubs in the left and right hemispheres, but that such connections do not result in a subject-specific global brain lateralization difference that favors one network over the other (i.e. left-brained or right-brained). Rather, lateralized brain networks appear to show local correlation across subjects with only weak changes from childhood into early adulthood and very small if any differences with gender.
